# Functionality and mechanistic parametric study of the potential of waste plantain peels and commercial bentonite for soybean oil refining

**DOI:** 10.1038/s41598-023-46842-1

**Published:** 2023-11-10

**Authors:** Annex Ifeanyi Ogbu, Prosper Eguono Ovuoraye, Regina Obiageli Ajemba, Mohammad Hadi Dehghani

**Affiliations:** 1 Department of Chemical Engineering, Nnamdi Azikiwe University, P.M.B. 5025, Awka, 420218, Nigeria; 2 Department of Chemical Engineering, Federal University of Petroleum Resources, P.M.B. 1221, Effurun, Nigeria; 3 Department of Environmental Health Engineering, School of Public Health, Tehran University of Medical Sciences, Tehran, Iran; 4 Center for Solid Waste Research, Institute for Environmental Research, Tehran University of Medical Sciences, Tehran, Iran

**Keywords:** Ecology, Environmental sciences

## Abstract

The consumption of unrefined vegetable oil poses acute and chronic health issues, yet improper disposal of waste plantain peels is not environmentally sustainable. This research investigates the feasibility, mechanism and thermodynamics of waste plantain peels, and commercial bentonite clay for soybean oil refining. Experiment was carried out using masses (1–4 g) of commercial bentonite clay, and unripe plantain peel ash (UPPA) to degummed and neutralized free fatty acid (FFA) contents in crude soybean oil at varying temperatures (50–120 °C), and time (15–35 min) for treatment of soybean oil. FTIR spectroscopy, SEM, and XRF techniques were applied to characterize the sample. The results established that at optimum 4.0 g dosage, the UPPA (97.73%) was more effective in the removal of FFA from oil at 50 °C and 20 min, while the clay (90%) was more effective in the removal of colour pigment from the vegetable oil 100 °C, and 25 min. The optimum efficiency of Clay-Ash-composite (70:30) in adsorbing pigment from soybean oil corresponds to 80%. The impact of changing viscosities, densities, and acid values on the performance of UPPA, clay, and clay-UPPA composite was investigated. Mechanistic studies confirmed the pseudo-second-order kinetics at 5 × 10^–2^ g/mg min^−1^ and 1.87 × 10^–1^ g/mg min^−1^, with corresponding adsorption capacity of 30.40 mg/g and 4.91 mg/g, at R^2^ ≤ 0.9982. The UPPA-driven sorption of FFA occurred as a physisorption and exothermic process (− 620.60 kJ/mol), while colour pigment removal occurred by chemisorption and endothermic process (22.40 kJ/mol). The finding recommends UPPA and composite as economically feasible for refining soybean oil.

## Introduction

Soybean is among the most widely grown oil crops worldwide. The kinds of margarine, salad dressings, cooking/frying oils, mayonnaise, and shortenings are the main products made with soya oil^1^. When crude edible oil is extracted from Soybean seed, it is known to contain soap residues, free fatty acids, phosphatides, trace metals, and colouring pigments^1,2^. The Soybean colour is caused by the presence of pigments in crude edible oil, such as chlorophyll-a, and B-carotene^2,3^. These substances degrade the oil’s quality by altering its colour and flavor, limiting its conservation and use^4^. Specifically, the presence of free fatty acid in soya bean oil affects the flavor, smoke point, oxidative stability, and durability of the oil^2^. Excess consumption of free fatty acid is detrimental to human health^4^, it causes insulin resistance, reduces glucose uptake in muscle, and increases glucose production in the liver, and can lead to hyperglycemia^5^. As a result, the importance of purifying Soybean oil to obtain a product with the desired colour and flavor cannot be overstated. Additionally, bleaching edible vegetable oil, such as soybean oil involves solely the removal of a variety of impurities like fatty acids, gums, trace metals, phosphatides, followed by decolourization, and it is one of the vital processes in industrial purification of vegetable oil to make it fit for consumption^4,6^. The refining process helps in raising their smoke point and storage durability and it is achieved industrially using natural or activated clays and activated carbons^1^.

The refining of soybean oil involves many stages carried out to improve its stability^7,8^, and remove unwanted impurities to make the oil ideal for cooking^8,9^. The degumming and neutralization stages are important for the removal of gum, and free fatty acids (FFA) compositions that might cause problems in the oil refining process^7,8^. Majority of research works applied the water and acid method of degumming vegetable oil^8^. The use of activated clays in bleaching edible oils has been investigated by many authors notably^10^, who investigated the optimum activation conditions of Ughelli bentonite clay for palm oil bleaching using response surface methodology^11^, examined the experimental process design for the sorption capacity of Kogi and Ibusa clay activated with HNO_3_ and H_2_SO_4_ in palm oil bleaching. Other researchers^12^ worked on the decolourization of palm oil by using Nigerian local clay^13^, and examined the chemical, mineralogical, structural, and surface properties^14^ of selected Ugandan clays and their application in bleaching oil.

According to^15^, Nigeria ranks as one of the highest producers and consumers of plantain in West Africa. The wastes generated from plantain contribute a lot to environmental hazards such as land pollution, air pollution, and water pollution, as they are usually disposed of carelessly, which may result in waterways blockage, and converting the town centers to foul-smelling scenes. In addition, unhealthy vegetable oil is unsafe for consumption^15^, reported that about 200,000 Nigerians dies annually from food poisoning and about 150,000 suffers from different heart disease, which all are traceable to high rate of consumption of unhealthy vegetable oil resulting from the high cost of refined vegetable oil. More so^15^, reasoned that an average Nigerian will opt for unrefined vegetable oil because the cost of refined vegetable oil is high. The bleaching process contributes significantly to the overall cost of refining vegetable oil because the commercially viable clay such as bentonite used for bleaching oil is expensive and it is imported from abroad^2,5^.

The application of waste plantain peels as catalysts have been reported in the literature as an adsorbent in wastewater treatment operations^9^, as a source of alkaline for solid soap production^16^, as a catalyst in biofuel synthesis^17^, and more recently the waste material has been applied for the reduction of carbon monoxide from biogas^18^ has been extensively carried out. However, the main advantage of the material is that it is available at no cost^9^. The previous research applied the principle of pyrolysis and calcination in preparation of the waste plantain peels at a high temperature of 600–700 °C to obtain active catalysts^17^, and absorbent materials^9,18^, and their results were reportedly feasible^16,18^. The use of unripe plantain peel ash (locally sourced) for bleaching vegetable oil, especially soya bean oil, will proffer a lasting solution to the high-cost of refined vegetable oil^7^ by automatically reducing the cost of refining the vegetable oil by substituting the commercial bentonite^9^ and at the same time reducing the environmental pollution through recycling of the unripe plantain peels. Yet, very limited research has been reported on the feasibility of waste plantain peels ash as a bleaching agent for vegetable oil; therefore, the bleaching capacity of ash materials produced from unripe plantain peel has not been thoroughly examined by researchers.

The present research explored the application of unripe plantain peel ash (UPPA) as an alternative to commercial bentonite for the bleaching of soybean oil (Soy oil). The physiochemical characteristics of unripe plantain peel ash were investigated following the Fourier transform infrared (FTIR) spectroscopy, scanning electron microscopy (SEM) analysis, and the X-ray fluorescence (XRF) technique respectively. The current work will also focus on the effect of bleaching parameters of UPPA and its adsorptive uptake of Free Fatty Acids (FFA), and pigment present in soya oil will be investigated and compared to that of commercially used bentonite. The statistically significant process parameters such as temperature, reaction time, and adsorbent dosage will be optimized. The effect of physiochemical parameters (viscosity, density, and acid value) on the bleaching and adsorption performance of UPPA was also studied and the outcome was compared with that of bentonite clay. The performance evaluation of the blending ratio of UPPA-clay composite was also examined. The mechanistic studies were carried out following the Freundlich and Langmuir isotherms, and the pseudo-first-order, and second-order kinetic models were employed to investigate the mechanism of removal of FFA and colour pigment from soybean. The thermodynamics evaluation was also surveyed to determine the feasibility of UPPA and its application in the vegetable oil refining process. It is believed that the information provided will aid decision-making regarding scale-up, and provide data for further research on the applicability of UPPA for commercial-scale refining of vegetable oil. The industrial-scale application of UPPA will reduce operational costs, and promote environmental sustainability through effective recycling and reuse of plantain bio-wastes. The research will also guarantee foster human health and safety through the availability of quality soybean oil stocks, human capital development through industrial scale production, and a quality environment via recycling of plantain wastes.

## Materials and method

### Material collection and sampling

The unripe plantain peel samples used for the experimentation were collected from a waste dump in a local farm located in Ijebu Igbo, Ogun State, Nigeria. The bentonite and crude soybean oil sample used in this experimentation were collected from the industrial facility of the Grand Cereals and Oil Mill Nigeria Limited. The author engaged in the batch-scale soybean oil refining process using standard analytical grade reagent which included sodium hydroxide 99%, distilled water 99.9%, methylated spirit 95%, phenolphthalein 98.5%, and n-hexane 97% purity respectively. All laboratory scale measurements and experimental methodology were performed independently, in compliance with guidelines set out by the Department of Chemical Engineering, Nnamdi Azikiwe University Awka, Anambra State, Nigeria.

### Preparation of the UPPA sample

The adsorbent was prepared from waste plantain peels disposed of its fresh and unripe plantains bunch that were harvested at maturity. The waste plantain peels weighing 80 kg were collected from a local farm located in the village of Ijebu Igbo in Ogun State, Nigeria (6.45° N, 4.00° E). The waste plantain peel sample was cleaned by washing with warm water at 30 °C to remove impurities and dried in an oven for three days at 110 °C to remove all volatile residue^9,16,17^. The waste plantain peel sample was ground with a blender to increase its surface area. The waste plantain peels were subjected to the calcination process in a muffle furnace 3L (MF2-12G) set at a temperature of 600 °C for 120 min following the methods reported in the published literature^7,8,17,20^. The purpose of the calcination treatment is to achieve the required chemical compositions and improved physical characteristics in the structure of waste plantain peel ash. The cleaning, physical activation, and calcination procedures were consistent with the methodology available in the literature^7,17,18,21^. The calcination process aids the decomposition and removal of volatile components present in the waste plantain peels^18,22^, and the physical activation provides the ash particle with the required energy to form bonds, improving its porosity and surface properties^9,21^. The weight of the waste plantain peel corresponding to 48 w/wt% after drying at 110 °C was recorded. After the calcination process, the ash derived from the waste plantain peels was weighed to obtain 17 w/w%^7,22^ of the peel as the approximate ash yield. The samples were passed through a 300-micron sieve to obtain uniform particle sizes of unripe plantain peel ash (UPPA).

### Characterization of the prepared UPPA sample

The prepared UPPA sample was characterized following the FTIR, SEM, and XRF techniques. The Fourier transform infrared spectroscopy (FT-IR) was applied to determine the functional groups present in the prepared UPPA sample using the IR-spectrophotometer model FTIR-8400S. Before analysis, samples were lightly grounded using a mortar and pestle and sieved again through the 150 µm mesh size. This was done to minimize the scattering, distortion, and peak broadening of IR radiation due to larger-sized particles that may have resulted from agglomeration during drying^2,4,17^. The spectra were recorded in the spectral range from 4000 to 750 cm^−1^.

The screened ash samples were separated into different particle sizes using standard sieves of mesh sizes. The X-ray fluorescence was performed on the prepared UPPA samples to determine the various chemical compositions that are present in the ash samples. An Oxford instruments model (ARL 9400XP+) wavelength dispersive XRF Spectrometer was used to perform the X-ray fluorescence analysis of the samples^9^. SEM analysis was carried out on the screened sample using a scanning electron probe micro-analyzer Zeiss (Perkin-Elmer, model spectrum 1, USA), to determine the surface morphology, porosity, crystalline, and grain structure of the prepared UPPA sample.

### Experimental procedure

**Figure 1:**
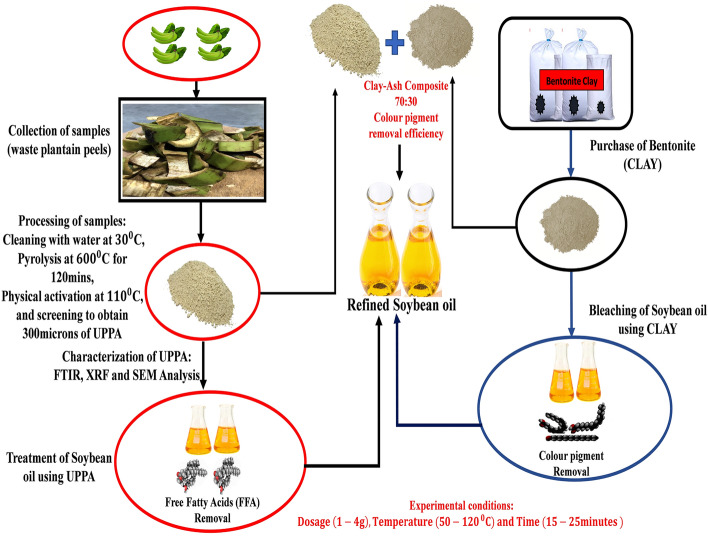
Flow diagram illustrating the summary of preparation and decolourization processes.

The collected soybean oil samples used in this experiment had been degummed and neutralized. Laboratory bleaching was performed in a 500 mL beaker equipped with a thermometer (TH300). The bleaching procedure was conducted using an electromagnetic hot plate and stirrer with an adjustable heater (A12-ES002). Firstly, an optimum bleaching time (20 min) and temperature (100 °C) were selected from the literature in accordance with previous literature^2,4^. 200 mL of the crude soybean oil sample was measured separately into four beakers labeled; 1 g, 2 g, 3 g, and 4 g respectively. The oil samples were gradually preheated up to 100 °C with constant mixing. The desired heating temperature (100 °C) was kept constant so that different masses (1 g, 2 g, 3 g, and 4 g) of the bleaching adsorbent (Clay) samples were weighed and added separately to the four beakers with the appropriate labels. The bleaching process was allowed to take place for 20 min, thereafter, the beakers were brought down and allowed to cool. The sludge was left to settle and was decanted into a filtration medium using filter paper^3^. The masses of the adsorbents were varied to test for the effect of dosage on the sample at a predetermined optimum of 20 min and 100 °C^2,3^. Different tests were carried out on the bleached samples and the optimum dosage sample was determined. The same procedure was repeated for UPPA using the same dosages varied from 1.0 to 4.0 g. The optimized dosage was then used in carrying out the bleaching process to allow for studying the effect of temperature and reaction time on the bleaching and adsorption process^3,4^. Using the optimized bleaching conditions obtained above, the effect of mixing the two adsorbents at different ratios was determined. Tests were carried out on the samples and results were recorded. The summary of the preparation and decolourization process is illustrated in Fig. 1 below.

### Characterization of the extracted soybean oil

American Oil Chemistry Society Standard (AOCS) procedures^23,24^ were employed to characterize the physical properties of the oil samples, which include viscosity, free fatty acid, density, specific gravity, colour removal, and acid value were consistent with reported literature^9^.

#### Viscosity measurement

The crude soybean oil sample was poured into an appropriate container until it touched the viscometer spindle’s oil mark. The speed of the spindle was set at 50 rpm. The control switch was turned on, and the spindle rotated in the oil for about 1 min for stability before taking the viscometer reading. The maximum reading of the viscometer was taken and recorded. The same procedure was carried out for all the bleached vegetable oil samples.

#### Density measurement

The density of the soybean oil sample was measured in the experiment using a 25 ml density bottle. An electric weighing balance was employed to measure the weight of the empty density bottle and the weight of the bottle filled with soybean oil. Based on the weight difference, the mass of soybean oil was determined. The density value was determined by evaluating the oil’s mass divided by its volume.

#### Determination of free fatty acid content

The oil was tested for acidity using the American Oil Chemistry Society method^23,24^. Firstly, 0.1N sodium hydroxide was prepared by dissolving 4 g of sodium hydroxide in 1.0 dm^3^ of distilled water and shaking vigorously. The freshly prepared solution was used to prepare a neutralized methylated spirit by titrating it against the methylated spirit with about 5 drops of phenolphthalein. Secondly, 25 g of the oil sample was weighed and placed in a dried conical flask. Approximately 50 ml of pre-neutralized methylated spirit was then added to the sample. Afterward, 5 drops of 1% phenolphthalein indicator were added to the mixture. The flask was subsequently positioned on a hot plate and heated until a temperature of around 40 °C was attained. The mixture was then titrated with a sodium hydroxide solution of 0.1 N until a pink colour change occurred for at least 30 s.

#### Absorbance determination

The absorbance measurements of the neutral (unbleached) and treated (bleached) oils were conducted on a UV–visible spectrophotometer (Model: UV-1800 Shimadzu UV Spectrophotometer) to evaluate the amount of pigment removed. The oil sample was diluted with n-hexane (1:4 v/v) and the bleaching capacity of the clay and ash was determined by measuring absorbance at 500 nm, which was the maximum absorptions observed in the wavelength range between 400 and 600 nm with the crude oil. The bleaching capacity was calculated by applying the Eq. 1 below^4, 25^:

1 $$\mathrm{Bleaching\, Capacity }=\frac{\mathrm{Absorbance\, of\, the\, untreated\, oil}\,-\mathrm{Absorbance \,of \,the\, treated \,oil}}\,{\mathrm{Absorbance \,of\, the\, untreated\, oil}} x 100$$

### Kinetics and isotherms theory

The Freundlich and Langmuir adsorption isotherm model, and the Pseudo-first-order, and Pseudo-second-order kinetics models were employed in this research work to investigate the kinetics, mechanism, and adsorption capacity of the UPPA and clay describes the soybean oil refining process.

#### Pseudo-first order

The pseudo-first-order kinetic model investigation of the bleaching process and the removal of the fatty acids from the Soybean oil was executed using the equation below^4,5,26^:

2 $$\mathrm{log}({q}_{e}-{q}_{t})=\mathrm{log}{q}_{e}-{k}_{1}t$$

3 $${q}_{\left(t\right)=\frac{{M}_{o}({C}_{i}-{C}_{t})}{{M}_{a}}}$$

4 $${q}_{\left(e\right)=\frac{{M}_{o}({C}_{i}-{C}_{e})}{{M}_{a}}}$$

where M_o_ is the quantity of oil (kg), M_a_ is the amount of adsorbent (kg), C_i_ is the initial adsorbate concentration at time t, and C_e_ is the adsorbate concentration at equilibrium e, usually the concentration is expressed in mgL^−1^ but in this study the concentration of the solution is equivalent to the pigment content in the oil, which in previous literature is expressed in basis weight^4,5,27^. The values of q_e_ and q_t_ are adsorption capacities at any time (q_t_) and in equilibrium (q_e_), k_1_ is the equilibrium rate constant of pseudo-first-order adsorption (gg^−1^ min^−1^). The slope of the plot of Log(q_e_ − q_t_) versus t was used to determine k_1_ (gg^−1^ min^−1^) and the intercept q_e_, q_(t),_ and q_(e)_ were determined^4,5,27^.

#### Pseudo-second order

The pseudo-second-order kinetic model investigation of the bleaching process can be expressed as following Eq. 5^4,5,28^:

5 $$\frac{t}{{q}_{t}}=\frac{1}{{k}_{2}{qe}^{2}}+\frac{t}{{q}_{e}}$$

where k_2_ (gg^−1^ min^−1^) is the equilibrium rate constant of pseudo-second-order adsorption^4,28^. The slope of the plot of t/q_t_ versus t was used to determine qe and then from the intercept, k_2_ was calculated. The rate constant was analyzed by employing Eq. 6^4^:

6 $$ln\frac{A}{{A}_{o}}=-k{(t)}^{0.5}$$

where A is the absorbance of the oil bleached at time t and A_0_ is the absorbance of crude or unbleached oil. From this equation, the linear regression between ln(A/A_0_) and t^0.5^ is a straight line whose slope is equal to − k^4^.

#### Freundlich isotherm model

7 $$log\frac{x}{m}=logk+nlog{X}_{e}$$

8 $$X=\frac{{A}_{o}-A}{{A}_{o}}$$

9 $${X}_{e}=1-X$$

where, x = the amount of substance adsorbed, m = the amount of adsorbent (bentonite clay), and Xe is the residual amount at equilibrium which is mathematically equal to (1 – X)^27,29^. The plot of log x/m versus Log Xe gives n as slope and log k as intercept^27,29,30^.

#### Langmuir isotherm model

10 $$\frac{{X_{e} }}{{x/m}} = \frac{1}{a} + \frac{b}{a}X_{e}$$

where a and b are Langmuir constant and saturated adsorption capacity. The plot of Xe/(x/m) versus Xe was used to evaluate the values of the slope and intercept corresponding to b/a and 1/a. From whence the values of the Langmuir constant K_L_ and adsorption intensity Q_e_ can be obtained^19,29^. To aid decision-making, the isotherms and kinetics model fits can be analyzed via statistical and error functions which include R^2^, and root mean square error (RMSE)^19,20^. For this research, the model fit statistics will be diagnosed using the RMSE error function, and $\Delta q$ outputs following Eqs. 11–12^19,20,31^.

11 $$\mathrm{RMSE }=\sqrt{\frac{1}{p-2}{\sum }_{i=1}^{p}qexp-qcal}$$

12 $$\Delta q\left(\text{\%}\right)=100\sqrt{\frac{\sum {\left|\left({q}_{exp}-{q}_{cal}\right)/{q}_{exp}\right|}^{2}}{N-1}}$$

where n and p correspond to the number of testing elements, and the number of parameters in the fitted model respectively. The value of q_cal_ is determined from model fit, and q_exp_ is computed from testing elements^19,31^.

### Adsorption thermodynamics

The thermodynamics of the adsorption process were determined using the set of Eqs. 13–15^4,29,32^ below:

13 $${K}_{d}=\frac{X}{{X}_{e}}$$

where X and X_e_ correspond to the amount of substance adsorbed, and the residual amount at equilibrium after treatment using UPPA and clay^4^.

14 $$In{K}_{d}= \frac{\Delta S}{R}-\frac{\Delta H}{R}\frac{1}{T}$$

15 $$\Delta G=\Delta H-T\Delta S$$

where $\Delta H$ is the enthalpy change of the system, T is the temperature (K), $\Delta G$ is the free energy of the system, and $\Delta S$ is the entropy of the system^14^. R is the molar gas constant^29,32^.

## Results and discussion

### Physiochemical properties of the soybean oil

Table 1 displays the characteristics of the oil before the refining process. The details show a low free fatty acid value (%FFA) of 0.926 which indicates that the soybean oil utilized is fresh^7,33^. Because free fatty acid is one of the undesired constituents that must be removed, its low percentage less than a unit will improve the efficiency of the refining process^8,26^. The acid value (1.852 mg/KOH/kg) and refractive index (1.47) of the oil sample are indications of the degree of oxidation and rancidity^25,34^. Consequently, when the life of soybean oil extends, the proportion of FFA increases due to hydrolysis in the presence of water and heat^35,36^. The density of the oil sample (907.2 kg/m^3^) suggests it slightly weighs less than an equal volume of water^6,26^. The soap residues, free fatty acids, phosphatides, trace metals, and colouring pigments are major impurities in crude edible oil produced from soybean seed^1,6^. These compounds reduce the quality of the oil by changing its colour and flavor, restricting its conservation and use. As a result, the significance of refining soybean oil to achieve the appropriate colour and flavor cannot be emphasized^7,8^.

**Table 1:** Physiochemical properties of the unbleached Soybean oil sample.

Parameters	Value
Absorbance	1.182
Specific gravity	0.9072
Density	907.2 kg/m^3^
%FFA	0.926
Refractive index	1.473
Acidic value	1.852 mg/KOH/kg

### Characteristic properties of the unripe plantain peel ash (UPPA)

#### XRF characterization

The X-ray refraction frequency (XRF) data (Table 2) revealed that the adsorbent (UPPA) is predominantly made up of the metallic oxides CaO, SiO_2_, Al_2_O_3_, Fe_2_O_3_, CuO, MnO, and ZnO. The highest percentage is in CaO (44.936), followed by SiO (35.448). The ZnO and CuO contents are present in very small quantities. The presence of these metal oxides is also clear evidence that unripe plantain peel ash will function as a potential catalyst^28,37^. Additionally, it can also be observed in Table 2 that, Si has the largest elemental composition, followed by Al and Ca, with trace amounts of Cu, Sr, Pb, Sb, and Ba. This outcome is consistent with the finding reported in the literature^38^. The presence of a lot of alumina and silica oxide in the ash suggests that it can be well configured for an excellent adsorbent^17,26^ and requires little to no chemical activation of its morphological characteristics^39^. It can be concluded that Si, Al, CaO, and SiO are the principal elements and metallic oxides that accounted for the adsorbent potentials of the UPPA^22,40^. Therefore, any chemical treatment might not be significant in the overall configuration of the biomass to prevent leaching of the key substituents resulting from mixing with carrier compounds^41,42^. The physical activation via reduction and screening of particle sizes is feasible, promotes reusability, and reduces the cost of the economy of sorbent regeneration^26,43,44^.

**Table 2:** Elemental and Oxide composition of UPPA X-Ray Fluorescence Cu–Zn Method.

Element	Composition (%)
Si	52.546
Ca	17.812
Al	27.356
S	0.967
Cl	0.260
K	0.234
Mn	0.257
Fe	0.205
Cu	0.023
Zn	0.143
Sr	0.052
Sb	0.02
Ba	0.026
Pb	0.007
Metalic oxide Composition	Ash content (%)
Cuo Fe_2_O_3_ MnO CaO Al_2_O_3_ ZnO SiO	3.03 5.29 0.33 44.94 10.17 0.18 35.45

#### SEM and FTIR analysis of the prepared UPPA

**Figure 2:**
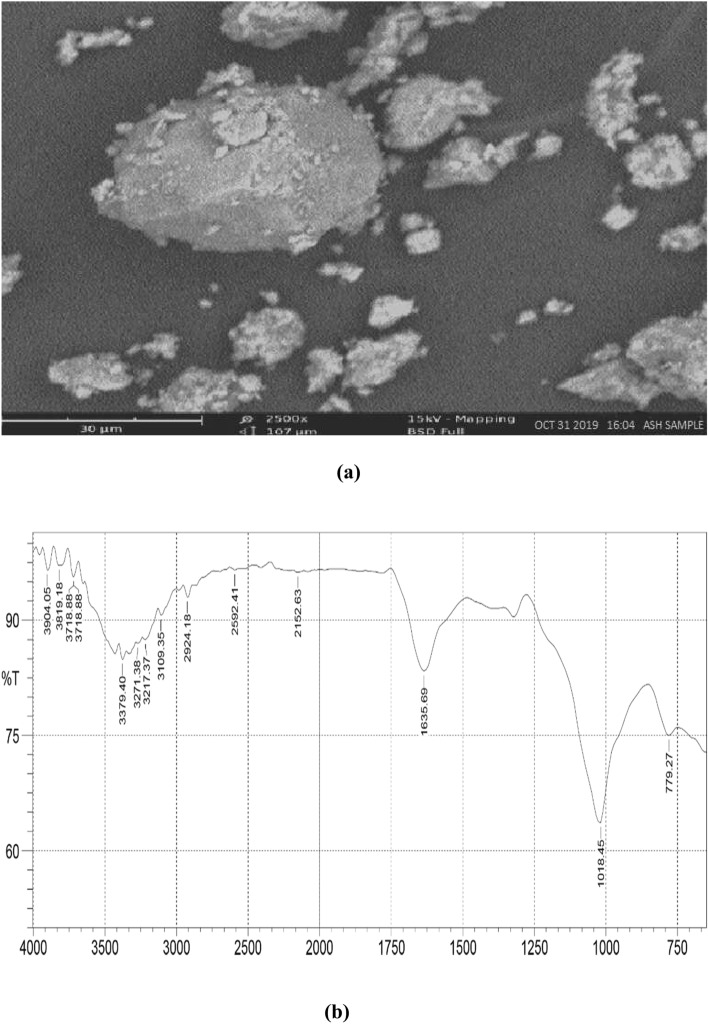
Characterization resulting (**a**) SEM image of Unripe plantain peel ash (UPPA), and (**b**) FTIR spectroscope of Unripe plantain peel ash (UPPA).

SEM examination on the UPPA shown in **2(a)** in Fig. 2 revealed the morphology of very fine-grained aggregates of ash platelets, irregularly curved flakes, and mats of coalesced flakes in the structure. In any case and because of particle coalescence, it is difficult to determine their exact texture. The UPPA was activated only by powdering it^7,17^. The ash consists mainly of large aggregates of nanoparticles and exhibits a distinct porous structure. The aggregate nature of the ash particle might limit the adsorption performance of the ash because the active site exposed is limited, and this outcome will possibly account for any discrepancies in adsorption property^9^.

The result of the IR spectrum on the prepared UPPA is shown in **2(b**) Fig. 2. The characteristics spectroscopy analysis indicates that smectite was the dominant mineral phase in the prepared ash sample. The characteristic IR bands between 3500 cm^−1^, 3904.05 cm^−1^, and near 3718.88 cm^−1^ indicate the presence of smectite. The absorbance peak formed at 3819.18 cm^−1^ is assigned to stretching vibrations of the O–H group of water molecules^45^. The broad band at 1635.69 cm^−1^ and the absorbance band at 1018.45 cm^−1^ to the OH bending vibration of sorbed water molecules present in the ash^9,17^. The presence of smectite in the ash further consolidates the result of XRF indicating that the unripe plantain peel ash is an effective adsorbent^31,40^.

### Effect of process parameters on bleaching and adsorption functionality

#### Influence of dosage of pure samples of UPPA and clay

**Figure 3:**
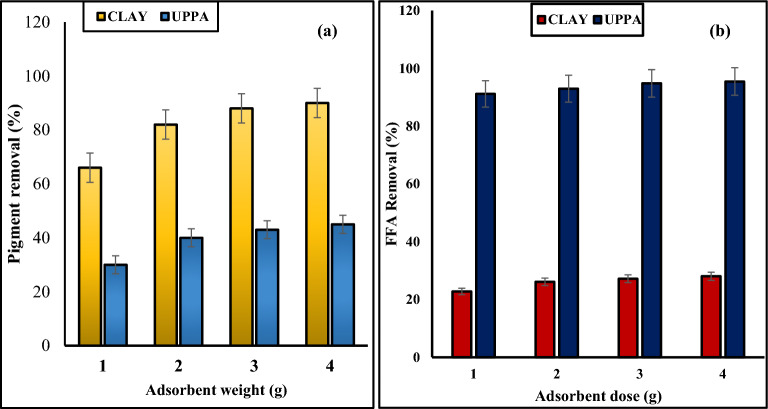
Effect of the dosage of functionality of pure samples of Clay and UPPA on; (**a**) Colour pigment removal and (**b**) Free fatty acid (FFA) removal.

It is challenging to determine the bare minimum amount of adsorbent required for efficient bleaching since different types of oil contain varying amounts of chemicals and respond in various ways to the adsorbent utilized. Consequently, the best amount of clay to use will depend on the type of clay and clay pretreatments, oil pretreatments, and desired oil grade^27,46^. The outline of Fig. 3a–b shows the bleaching functionality of soybean oil with either adsorbent (clay and ash). The number of colour pigments and FFA content of the soybean oil was reduced as the dosage of UPPA and clay increased from 1.0 to 4.0 g^47^. The outcome suggests the removal of unwanted components from the vegetable oil is improved by higher adsorbent weight^5^. The maximum percentage of colour pigment removed corresponds to 45 and 90% for UPPA and bentonite clay, respectively. While the optimum FFA adsorption efficiency corresponds to 28.07 and 95.50% for clay and UPPA, respectively. The outcome of Fig. 3 demonstrates that an optimum 4.0 g dosage recorded for refining soybean oil has a significant effect on the performance of ash produced and bentonite clay. Although, the pigment absorbance property of UPPA was poor (44.76%). It can be concluded that the ash (UPPA) produced was more efficient for FFA removal than commercial bentonite clay. This outcome suggests UPPA has a higher affinity for FFA than colour pigment^3,48^ whereas pigment is strongly bound by the bentonite clay at a higher dosage^10,48^. An increase in the active site available for adsorption accounts for the increase in bleaching power with the adsorbent weight^10^.The statistical test result exercised on the experimental data proved that the dosage has trivial antagonistic effect on the sorption of FFA and pigment from soybean oil onto UPPA and clay. The test statistics confirmed dosage was not significant on the overall performance of the adsorbent at *p*-values > 0.05 and 95% confidence interval^39^.

#### Effect of contact time of pure samples ash and clay

**Figure 4:**
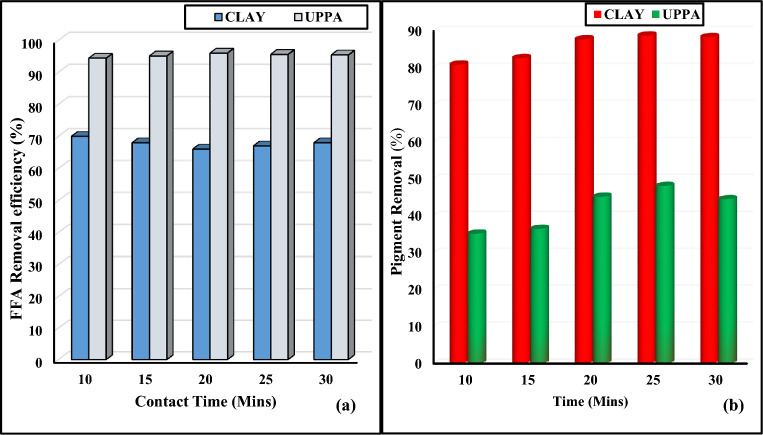
Contact time of Pure samples UPPA and Bentonite clay on; (**a**) FFA removal and (**b**) Pigment removal.

The best bleaching performance of biosorbents is determined by the bleaching time, temperature, and adsorbent quality^10^. Colour removal rises with time and temperature, but prolonged contact between oil and clay might result in colour reversion, which also increases with temperature^2,36,47^. The plot of Fig. 4a–b shows that bleaching capacity increases from 10 to 25 min and starts decreasing from 25 to 30 min for both UPPA and the clay. The proportion of discolouration rises as contact time increases. The highest colour removal efficiency of both adsorbents was recorded within 20–25 min. This finding is consistent with that of^2,29^, who reported that the ideal contact time for bleaching soybean oil is between 20 and 25 min. Therefore, we can state that desorption (colour reversal) occurs at any contact duration longer than 25 min. In addition, the outcome of Fig. 4a demonstrates that ash adsorbed more FFA than the clay with maximum adsorption efficiency of 95.68% occurring at optimum 20 min. At an optimum contact time of 25 min, the bentonite clay performed better than UPPA with pigment removal efficiency transcending to 88.70 and 48.01% for clay and ash respectively. The outline of Fig. 4b confirmed that the active sites on UPPA became saturated with adsorbed pigment and reached their maximum efficiency (48.01%) at 25 min, where a further increase in contact time led to a decrease in removal efficiency^8^. The statistics analysis at a 95% confidence interval confirmed that contact time has a significant effect on the removal of FFA, and colour pigment from the oil medium at a *p*-value of 0.04 and 0.005 for UPPA and clay, respectively. The effect of contact time on the UPPA-driven sorption of colour pigment from oil was not significant at *p*-value > 0.05^39^.

#### Effect of temperature on pure samples of ash and clay

**Figure 5:**
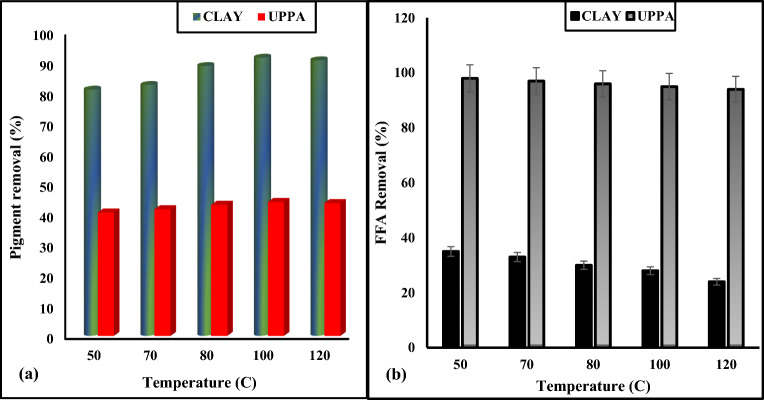
Effect of Temperature of Pure samples UPPA and Bentonite Clay on; (**a**) Pigment removal and (**b**) FFA removal.

According to^2,32,49^ the type of oil and amounts of colour bodies, oxidation products, and impurities influence the optimal bleaching temperature. In Fig. 5a, the bleaching effectiveness or percentage of colour pigment removal increased intermittently as the temperature rises from 50 to 120 °C. This was probably due to the increase in thermal energy, which increased the activity of the clay surface and the diffusion rate of the colour pigment^4^. The percentage of colour removal reaches its maximum (92 and 44.2%) at 100 °C, for clay and ash respectively. The optimum performance is consistent comparable to the published reports of^7,25,28^ having 100 °C, 95 °C, and 96 °C respectively as the optimum bleaching temperature for pigment adsorption from soybean oil. In addition^50^, carried out a comparative study on the adsorption of FFA present is soybean oil using Aluminum Oxide, Rice Husk Silicate (RHS), and Rice Husk Ash (RSA), the result showed that aluminum oxide has the highest adsorption efficiency of 40%. Also^51^, obtained 90% FFA removal from soybean oil using Ca(OH)_2_ nanoplates supported on activated carbon. Comparing these outcomes to the present study proved that UPPA outperformed the adsorbents investigated in FFA adsorption from soybean oil at optimum temperature of 50 °C. The better performance of clay was probably due to the hydrolysis reaction taking place in the oil as a result of heat^36,52^, which may also lead to swelling of clay particles, exposing more surface area and increasing the adsorption capacity of bentonite. The percentage of colour removal of both adsorbents drops to 90% and 43%, at 120 °C. This outcome was due to a degradation of clay structure and probable loss of its adsorption capacity^26^. In addition, the adsorbate-adsorbent complex, which forms as temperature rises and becomes unstable may be the reason for the decline in adsorption^40,43^. This result agrees with the published work of^29,52^. According to previous literature, the ideal bleaching temperature varies depending on the adsorbent and oil, and for palm oil, it is between 100 and 120 °C to^30,52,53^. Also, bleaching at high temperatures for an extended period has a negative impact on the oxidative stability of edible oil^2,54^. Furthermore, the outline of Fig. 5b confirmed the free fatty acid content in soybean oil decreased significantly as temperature increased from 50 to 120 °C. At the optimum temperature (50 °C), UPPA recorded a significantly higher FFA adsorption efficiency of 97.73%, while 35.10% FFA removal efficiency was recorded for clay. This result confirmed that the UPPA becomes saturated at a temperature > 50 °C, and beyond this optimum the adsorbent may start to re-release some of the previously adsorbed FFA back into the oil medium, resulting in a decrease in removal efficiency at elevated temperature^29,48^. The test statistical analysis proved that temperature has a significant antagonistic effect on the colour removal efficiency of the clay at a *p*-value of 0.005, bet the temperature has no significant effect on ash-driven sorption of FFA from the vegetable oil at a 95% confidence level^39^.

#### Impact of physicochemical properties on the overall performance of adsorbent

**Figure 6:**
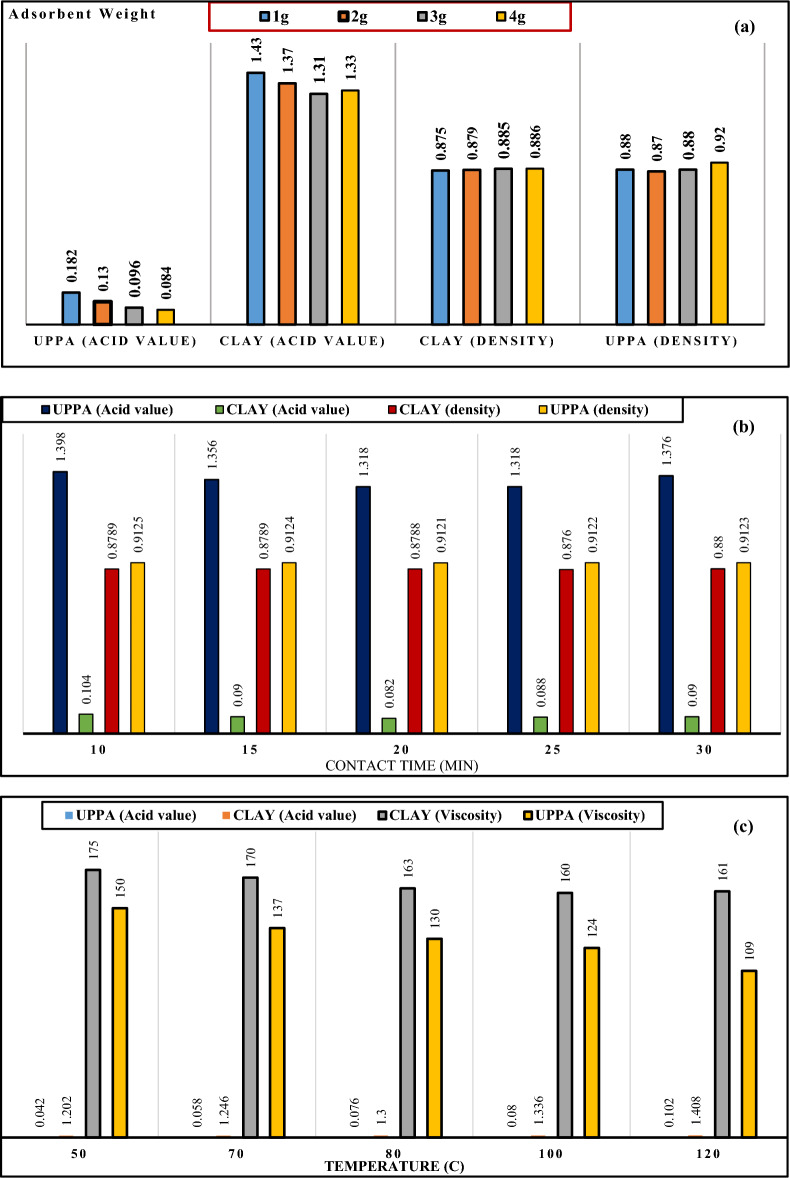
Effect of the physiochemical properties of UPPA and Clay samples on performance functionality at; (**a**) Dosage (**b**) varying contact time, and (**c**) Temperature.

The outlay of the bar chart in Fig. 6a confirmed that the acid value and density of soybean oil decreased as adsorbent doses increased from 1.0 to 4.0 g. The minimal acid values (0.083 and 1.33), and density values (0.886 and 0.93) were recorded at optimum dosage (4 g) that transcends to maximum FFA and pigment removal. The outcome proved that at higher dosages, there is a greater surface area/sites available for adsorption to occur^22,40^. Consequently, more amount of FFA, colour, and other impurities bind with the active sites resulting in higher efficiency of UPPA^4,5,29^.

The findings from the bar chart in Fig. 6b proved that the density, and acid value of the soybean oil decreased with reaction time from 10 to 25 min. The decrease in density and acid value of the oil corresponds to better FFA and pigment adsorption to clay and ash^49^. The UPPA surface becomes saturated with free fatty acid^22,31^ at contact time > 25 min, and can no longer bind free fatty acid molecules to its surface causing efficiency to drop to ≤ 96%.

The bar chart in Fig. 6c showed a steady reduction in the viscosity of soybean oil as temperature increased from 50 to 120 °C. This outcome suggests a decrease in surface tension due to a decrease in viscosity, which hindered the attraction of pigment molecules and diffusion unto UPPA yielding ≤ 44.2% colour pigment removal. However, the decrease in viscosity of the oil with temperature rise resulted in better dispersion of pigment particles, improved clay oil interactions, and flowability^32,49,52^ transcending to 91% pigment removal. The molecular interaction of FFA and oil molecules weakens at higher temperatures^7^. Consequently, maximum FFA removal was recorded at optimum 50 °C, where the acid value (0.042) and density (169) of soybean oil is minimum. The optimum temperature is consistent and agrees with the sorption performance of using plantain extract as an adsorbent^55^. It can thus be concluded that the application of UPPA greatly favored the removal of free fatty acid from soybean oil compared to bentonite clay. Whereas the clay yielded superior performance over the ash concerning pigment removal from soybean oil. Consequently, an investigation into clay-UPPA composite for refining soybean oil becomes imperative.

#### Effect of mixing ratio on performance clay-ash composite

**Figure 7:**
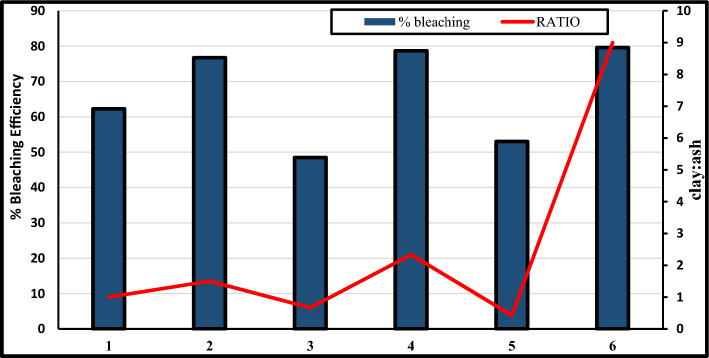
Effect of blending ratio on bleaching performance of the clay-ash composite.

The outline of Fig. 7 illustrates that increasing the clay-to-ash ratio improves the bleaching efficiency of the composite, with the highest ratio of 9:1 having the highest bleaching efficiency of 79.61%. The pigment removal efficiency of clay-ash composite varies intermittently as the blending ratio changes. A blending ratio of clay-ash of 1 yielded a minimum pigment removal efficiency of 34.38% as shown in Table 3. The finding confirmed that the more the proportion of bleaching clay used, the higher the percentage of colour removal and the lower the amount of free fatty acid removed from the crude Soybean oil^8^. This output also established that the clay is more efficient than the ash at removing pigment while the ash absorbed more free fatty acid than the clay^7,8^. Consequently, an optimum clay-ash composite of proportion 7:3 will stabilize the pigment removal efficiency of UPPA in the soybean oil system, configure the density of the composite to 0.8996, reduce the acid value of oil, and drive the performance towards $<$ 80%.

**Table 3:** Effect of mixture of pure samples of UPPA and clay on bleaching.

CLAY: UPPA	FFA	Acid value	Absorbance	Percentage colour removal	Viscosity	Density
50:50	0.0564	0.1128	0.446	62.2673	160	0.8988
60:40	0.0664	0.1328	0.275	76.7343	144	0.8992
40:60	0.0500	0.100	0.609	48.4772	126	0.8992
70:30	0.0902	0.1804	0.252	78.6802	79.8	0.8996
30:70	0.0464	0.0464	0.555	53.0457	71.4	0.9032
90:10	0.3835	0.767	0.241	79.6108	150	0.9016
10:90	0.0338	0.0676	0.752	36.3790	153	0.9012

### Kinetics of adsorption and bleaching process

**Figure 8:**
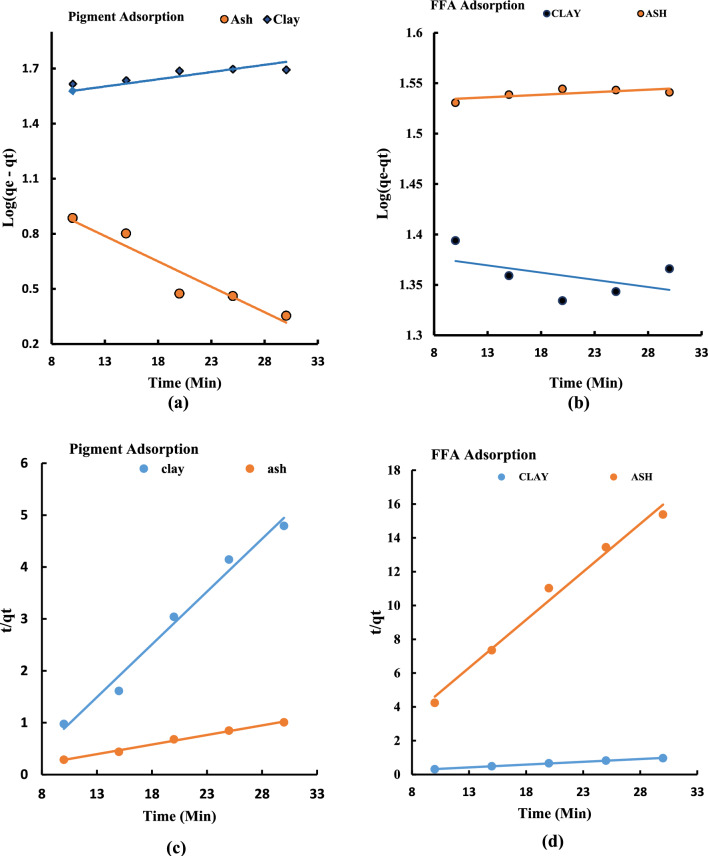
Kinetic traces of UPPA and Bentonite clay showing (**a**) Pseudo-first-order Pigment adsorption (**b**) Pseudo-first-order FFA adsorption (**c**) Pseudo-second-order Pigment adsorption (**d**) Pseudo-second-order FFA adsorption.

Figure 8a–b shows the respective kinetic traces for the pseudo-first-order and pseudo-second-order sorption design for the soybean refining process at optimum condition of temperature. The linear plot of log (qe–qt) against t in Fig. 8a–b gives a slope and an intercept from whence the value of pseudo-first-order rate constant k_1_ was determined. The value of pseudo-first-order constant k_1_ for the UPPA-driven adsorptive uptake of colour pigment was evaluated to be equal to 2.81 × 10^–3^ min^−1^ and the amount of colour pigment adsorbed at equilibrium qe was evaluated to be equal to 14.40 mg/g at the coefficient of determination, R^2^ equal to 0.9055. For the clay-driven pigment adsorption, the values of k_1_ and q_e_ were recorded to be equal to 4.3 × 10^–3^ min^−1^ and 38.10 mg/g respectively at R^2^ (0.8331). For the UPPA-driven removal of FFA from soybean oil the value of pseudo-first-order constant k_1_ = 5 × 10^–4^ min^−1^ and qe = 33.83 mg/g was recorded with an R^2^ value of 0.9434. While the pseudo-first-order constant for the clay-driven removal of FFA was evaluated to be k_1_ = 1.4 × 10^–3^ min^−1^ and qe (24.43 mg/g) was recorded with an (R^2^) value of 0.8242 as shown in Table 4.

**Table 4:** Kinetics and isotherm parameters for UPPA/Clay-driven sorption of FFA and Pigment sorption at optimum condition.

Freundlich model		Langmuir model		Pseudo-first order		Pseudo-second order	
(CLAY) Pigment adsorption at optimum 100 °C							
K_F_	1.70	K_L_	0.15	K_1_	4.3 × 10^–3^	K_2_	3.5 × 10^–2^
n	3.99	q_max_	0.91				
R^2^	0.9302	R^2^	0.9041	R^2^	0.8331	R^2^	0.9833
$\Delta q\left({\%}\right)$	15.17	$\Delta q\left({\%}\right)$	9.22	q_cal_	38.10	qcal	4.92
RMSE	3.83	RMSE	0.41	–	–	–	–
–	–	R_L_	0.74	–	–	–	–
(UPPA) Sorption of free fatty acid (FFA) at optimum 50 °C							
K_F_	59.40	K_L_	10.35	K_1_	5 × 10^–4^	K_2_	1.87 × 10^–1^
n	17.96	q_max_	1.11	–	–	–	
R^2^	0.9969	R^2^	0.9941	R^2^	0.9434	R^2^	0.9986
$\Delta q\left({\%}\right)$	50.10	$\Delta q\left({\%}\right)$	20.73	q_e_	33.83	q_e_	30.40
RMSE	7.60	RMSE	0.61	–	–	–	–
–	–	R_L_	0.90	–	–	–	–

The adsorption results from Table 4 show that the values of the pseudo-second-order rate constant of adsorption for Clay and UPPA are evaluated from the slope and intercept of the kinetics trace in Fig. 8c–d. The results confirmed that the value of k_2_ = 3.5 × 10^–2^ g/mg min and q_e_ = 4.92 mg/g respectively, were recorded for pigment adsorption to bentonite clay. This output transcends to the coefficient of determination, the R^2^ value of 0.9833. For the UPPA-driven sorption of colour pigment from soybean oil corresponds to k_2_ = 1.58 × 10^–2^ g/mg min and q_e_ = 27.10 mg/g respectively, recorded with an R^2^ value of 0.9945. For the FFA adsorption process, the values of k_2_ (1.87 × 10^–1^) and q_e_ (30.40 mg/g) were obtained at an R^2^ (0.9986) for UPPA, while k_2_ (3.0 × 10^–1^ g/mg min) and q_e_ (1.76 mg/g) respectively, following a coefficient of determination R^2^ value of 0.9862 was recorded for the clay sorption of FFA. Studies have shown that a good kinetic model must have a good correlation coefficient for data fit closest to a unit (R^2^ ≤ 1)^14,18^ and the value of q_e_ must be close to the experimental value^34,56^. Therefore, the Pseudo second-order model is more applicable in this experiment because the values of R^2^ recorded for both ash and clay are greater than the R^2^ values obtained from the Pseudo first-order model, and the calculated value of q_e_ was closer to the experimental value. This outcome is consistent and agrees with the findings reported by^10,57,58^.

It can be concluded from the kinetics result that the adsorption data obtained from the soybean refining experimentation best suit the pseudo-second-order sorption kinetics. The result indicates that the rate at which adsorption of free fatty acid and pigment was proportional to the square of the amount of unreacted adsorbate sites on UPPA and clay. The equilibrium was attained faster than the first-order at 1.87 × 10^–1^ g/mg-min, suggesting a rapid transfer of FFA molecules onto the UPPA surface at a relatively high capacity (33.83 mg/g) under the optimum condition studied. The initial rate of sorption of colour pigment from the vegetable oil environment is significantly higher in bentonite clay compared to ash. This development was probably due to differences in sorption mechanisms^4^, and chemical configurations of the adsorbent material which account for the mass transfer resistance in the soybean oil system^5,10^.

### Adsorption mechanism and isotherm of the pigment and FFA sorption process

**Figure 9:**
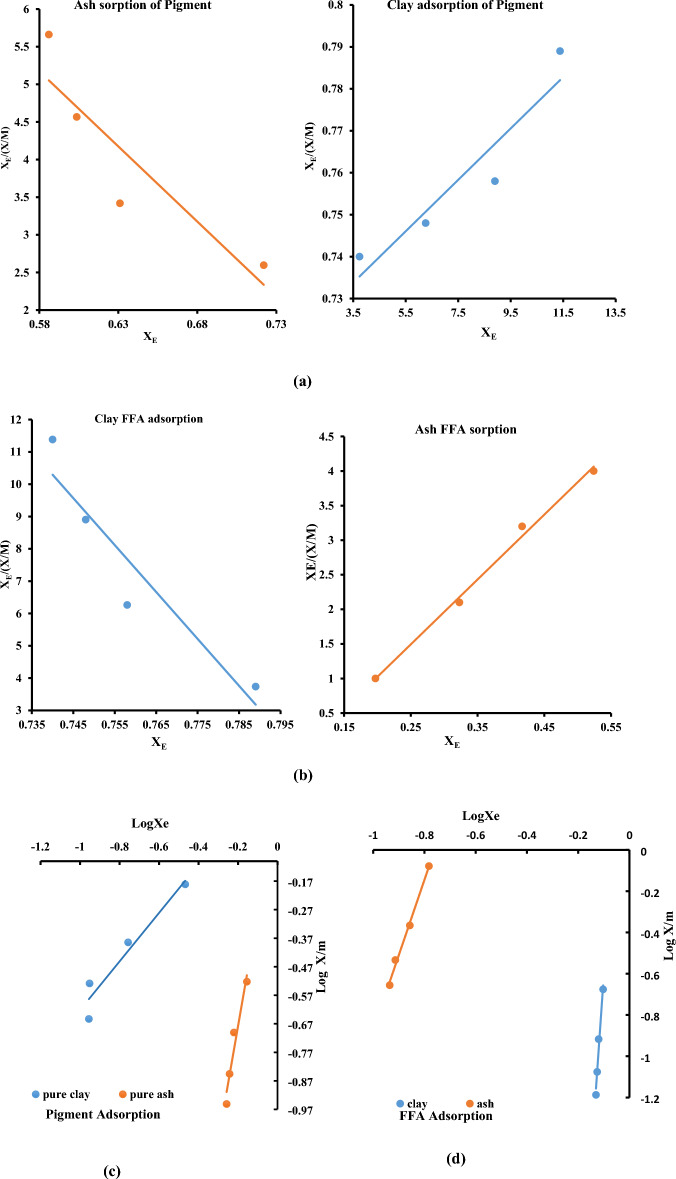
Isotherm traces showing (**a**) the Langmuir model for pigment adsorption unto ash and clay, and (**b**) Langmuir model for the FFA adsorption unto ash and clay, (**c**) the Freundlich model for the pigment adsorption unto ash and clay, (**d**) the Freundlich model for the FFA adsorption onto ash and clay.

In the current research, two isotherm models were employed for the interpretation of the mechanism of adsorption of free fatty acid (FFA) and colour pigment onto Ash and Clay. The isotherm results were obtained from the isotherm traces (a–d) in Fig. 9. The outputs are summarized in Table 4**.** The value of Langmuir constant K_L_ defines the activity of the adsorbent for a particular solute^25^, and the maximum sorption constant (q_max_) accounts for the saturated adsorptive capacity of the adsorbent. A negative value of the slope (− 15.729) and an intercept of 13.11 was obtained for the ash-driven sorption of pigment from soybean oil at R^2^ (0.8141). Consequently, the Ash-driven sorption of pigment from the soybean oil is inversely proportional to surface coverage leading to a saturation active site of ash^4,32^. This outcome accounts for the moderate pigment removal efficiency of $<50\%$ reported for the UPPA system^3,47^. The result suggests that the UPPA may not be an effective adsorbent for colour pigment at optimized conditions. Comparatively, the best performance of the commercial bentonite (clay) in the sorption of colour pigment established Langmuir constant K_L_ (0.15 Lmol^−1^) with a corresponding 90% efficiency, and maximum adsorption capacity q_max_ (0.91 mg/g) recorded at R^2^ (0.9041). For clay-driven the FFA sorption process, a negative value of slope (− 0.0061) and an intercept (0.8052) was also recorded with an R^2^ value of 0.9012. These outputs account for the low performance $<35\%$ reported for the clay-driven sorption of FFA from soybean oil. Consequently, the finding established that bentonite (clay) was not effective for the removal of free fatty acid from the vegetable oil at the optimized conditions^32,48^. However, a Langmuir constant (K_L_) of 10.35 Lmol^−1^ described the sorption intensity of the UPPA-FFA sorption system with an efficiency > 97%, and corresponding monolayer adsorption capacity (q_max_) of 1.11 mg/g obtained with an R^2^ value of 0.9969. Consequently, it can be inferred from the Langmuir isotherm result that the bentonite clay is more effective in adsorbing pigment from soybean oil, while the UPPA is more efficient in adsorbing FFA from the vegetable oil. The finding recorded was in reasonable agreement with the published work reported in published literature^47^. The values of the separation factor (R_L_) recorded following the mechanism of the colour pigment, and FFA sorption from soybean oil translate to 0.90 for the UPPA, and 0.74 for bentonite clay respectively. These values of R_L_ were consistent > 0 and $<$ 1, indicating the respective adsorption of FFA and colour pigment onto UPPA and bentonite clay were both favorable^5,19,32^. Thus, suggesting that the heat of adsorption does not depend on the number of sites and is equal for all sites^19^.

The Freundlich isotherm parameters are presented in Table 4. The results show values of n which is related to the bonding energy of adsorption of pigment molecules to the be adsorbents corresponding to 3.99 1/mg and 0.85 1/mg for clay and UPPA respectively. The respective values of n which describes the bonding energy of the sorption of FFA molecules to the active surfaces were found to be 3.65 1/mg and 17.96 1/mg for bentonite clay, and UPPA. The result confirmed values of n > 1.0, indicating that the adsorbents are effective for removal of colour pigment and FFA contents in the vegetable oil refining process^48,56,59^. For a value of n less than 5 the adsorbent might be effective for reducing the initial amounts of colour but less effective for achieving the maximal degree of bleaching than if the value of n is high^59^. Higher values of empirical coefficients n = 17.96 l/mg recorded for the sorption of FFA to the active surface of UPPA, and n = 3.98 l/mg recorded for clay (bentonite) are consistent and agree with the report from published works available in the literature^12,56^. Additionally, the value of Freundlich constant (K_F_) defines the activity of the adsorbent for a particular solute^25^. In this case, it is used to describe the adsorption of colour pigment and FFA onto the surface of bentonite clay, and UPPA respectively. The average values of Freundlich constant "K_F_" were estimated to be 1.31 mg/g and 1.70 mg/g respectively, in pigment adsorption unto UPPA and clay. While value of K_F_ that defines the FFA sorption from the soybean oil unto ash and clay systems correspond to 59.40 mg/g and 15.65 mg/g. These outcomes transcend to the model's coefficient of determination, R^2^ values which confirmed the data fit the model to be equal to 0.9302 for clay and 0.9420 for UPPA in pigment adsorption, 0.9941 and 0.9729 respectively, for FFA removal in ash and clay system. The average value of K_F_ = 59.40 mg/g recorded for the unripe plantain ash (UPPA) is indicative of a relatively high adsorption capacity of the adsorbent for FFA, while the higher values of K_F_ = 1.70 mg/g recorded for clay (bentonite) agrees with the Langmuir assumption that a relatively high pigment sorption capacity is predominant for the clay.

The results from the statistical error function exercised to describe the Langmuir isotherm description mechanism of the removal of FFA from the soybean oil showed that the RMSE (0.61), and $\Delta q(\%)$ equal to 20.73 respectively with an R^2^ (0.9969) were recorded for the sorption of FFA to UPPA. While an RMSE value of 7.60 and $\Delta q(\%)$ value of 50.10 with a corresponding R^2^ (0.9941) for the Freundlich isotherm description of sorption of FFA from soybean oil unto the ash. The findings established that the Langmuir isotherm model yielded lower error statistics with a higher correlation coefficient description of model fit for the UPPA-driven sorption data^19^. Furthermore, RMSE, $\Delta q(\%)$, and R^2^ values of 3.83, 9.20, and 0.9041 were recorded for the Langmuir description of the adsorption of colour pigment unto the clay. While an RMSE, $\Delta q(\%)$, and R^2^ values of 0.41, 15.17, and 0.9302 were recorded for the Freundlich isotherm description of the sorption of colour pigment from vegetable oil to bentonite clay. Comparatively, the statistical evaluation metrics proved that lower statistical errors were associated with the Freundlich isotherm model relative to the values recorded for the Freundlich isotherm. The Freundlich isotherm fitted well for both the Ash and Clay in the pigment sorption process because the R^2^ obtained in the Freundlich isotherms is higher^26,39^, with significantly lower statistical error evaluation metrics than those obtained in the Langmuir isotherm. This supports the findings ‘that the Freundlich isotherm applies better to liquid phases while the Langmuir isotherm applies better to gaseous phases^35,60^.

Overall, it can be concluded that the clay surface was uniform and independent of each other and allowed for multiple adsorptions by colour pigment that led to a solid–liquid equilibrium to be established. The Langmuir isotherm model proved to be superior in Ash's FFA sorption process, indicating that adsorption happened uniformly at specific sites with equal energy on the surface. The UPPA-driven sorption of FFA occurred via a single-step process with negligible interaction between absorbed molecules^5,32^. Insufficient FFA concentration for surface saturation causes adsorbed molecules to remain on ash surfaces without dissociation.

### Thermodynamics of the soybean oil refining process

**Figure 10:**
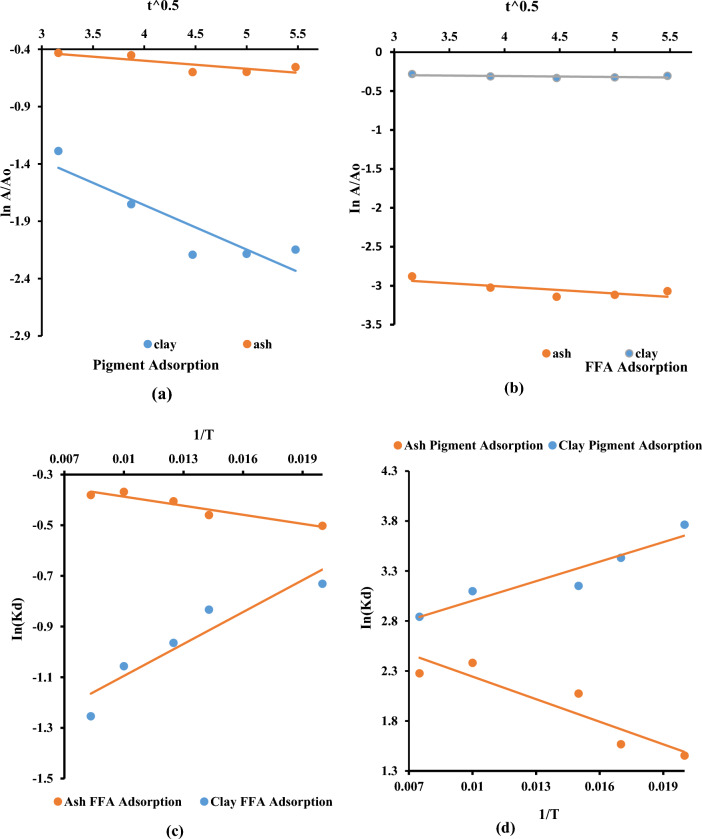
Thermodynamics traces of the soybean oil refining process for determining; (**a**) Constant for pigment sorption (**b**) Constant for sorption of FFA (**c**) Parameters for UPPA and Clay adsorption of FFA, (**d**) UPPA and Clay adsorption of colour Pigment.

The thermodynamics modeling of the UPPA-driven sorption of FFA and adsorptive uptake of colour pigment from soybean oil onto clay was surveyed to create a better interpretation of the bleaching and soybean oil refining process at optimum condition. Typically, the heat developed or absorbed during the adsorption process is referred to as the heat of adsorption^2,4,5^ which was determined from the analysis of the plots in Fig. 10a–d. The plot of lnA/Ao against t^0.5^ shown in Fig. 10a–b was used to determine the values of rate constants (k), which were found to be 7.05 × 10^–2^ for ash and 3.88 × 10^–1^ for clay in pigment adsorption, while in the FFA adsorption, the rate constants were 8.77 × 10^–2^ and 1.23 × 10^–2^ for ash and clay, respectively. The finding proved that the rate of adsorption increases as k increases, as a result the clay adsorbs colour pigment from the soybean oil more readily than the ash, while the ash adsorbs FFA efficiently than the clay. This result agrees with the findings reported in the works of^48,61^.

However, the exothermic nature of the adsorption process evaluated from Fig. 10c–d is indicated by the negative value of the heat of adsorption ($\Delta H$ ) of − 42.08 kJ/mol recorded for the UPPA-driven sorption of colour pigment, and − 620.60 kJ/mol for the sorption of FFA from soybean oil. The heat of adsorption for clay (bentonite) was found to be 22.42 kJ/mol for pigment and − 356.37 kJ/mol for the sorption of FFA from soybean oil respectively. The outcome suggests that clay's adsorption mechanism for pigment is endothermic, while that of the UPPA-FFA system is exothermic^4,32^. Furthermore, the UPPA-driven uptake of FFA is exothermic, while that of clay was exothermic. The endothermic sorption process of the clay is in contrast to the findings obtained by reference^27^ while the exothermic sorption process of UPPA conformed to the outcome of^27^. The results indicate that adsorptive uptake of FFA from the vegetable oil occurred when the heat of adsorption is less than 20 kJ mol^−1^ and the adsorption process for the removal of colour pigments from soybean oil occurred at energy complex > 20 KJ mol^−1^^58,59^. The finding is consistent with pigment sorption characteristics of clay reported in literature^33,58,62^. Following the thermodynamics relation evaluated from analysis of Eqs. 14–15^4^ that, a Gibb’s free energy $(\Delta G)$ of − 7068.33 kJ/mol, with an entropy change ($\Delta S)$ corresponding to 19.01 J/molK, where recorded for the clay-driven sorption of colour pigment from soybean oil. This outcome confirmed the removal of colour pigment from soybean oil was a spontaneous process and requires the arrangement of molecules^4,5^, which increases the disorder of the clay-pigment system^32^. However, a positive value of Gibb’s free energy (30,184.98 kJ/mol) suggests that the adsorptive uptake of colour pigment from the soybean oil is non-spontaneous, unfavorable, and occurred at a constant temperature (50 °C)^2,3^. While the negative value of $\Delta G=$− 6761 kJ/mol recorded for the UPPA-driven adsorptive uptake of FFA suggests that the process is favorable and spontaneous^4,5,32^. The negative entropy (− 11.78 J/mol.K) indicates a decrease in the degree of disorderliness for the system due to strong bond energy^55^, and that the active sites on the ash have a high selectivity and affinity for free fatty acids^32,48^. Consequently, the Ash-driven sorption of FFA from the vegetable oil becomes more orderly, and the sorption process reached equilibrium after 20 min. This information will be important in designing processes for the removal of FFA from vegetable oil. It can be concluded from the thermodynamics that the clay adsorbs the soybean oil's colour faster than the ash while the ash adsorbs FFA faster than the clay.

## Conclusions

The study investigated the effectiveness of Unripe Plantain Peel Ash (UPPA) as a substitute for commercial bentonite (clay) in refining soybean oil. The study also analyzed how temperature, dosage, and contact time impacted the outcome. It was discovered that as temperature, time, and dosage increase, so does FFA removal efficiency, while an increase in temperature increases the amount of colour removal in the soybean oil system. The findings proved that UPPA is more effective than clay in adsorbing the free fatty acid in soybean oil, whereas commercial bentonite is more effective in adsorbing the pigment in soybean oil. The optimum performances transcend to 97.73% removal efficiency for UPPA-driven sorption of FFA from soybean oil at 50 °C, using 4 g dosage, and requiring 20 min of contact time. A 90% pigment removal efficiency was recorded using 4 g of bentonite clay at 100 °C, and 25 min of contact time. Results from the mechanistic studies on the adsorption process confirmed that the soybean oil refining process followed pseudo-second-order kinetics, while the rate of soybean oil pigment adsorption by clay fits well with the Freundlich isotherm. The rate of FFA adsorption by UPPA in the soybean oil system proved to be viable, with greater commercial feasibility than the bentonite clay used in the industry. The thermodynamics survey confirmed that the vegetable oil refining process is dependent on kinetics. The removal of FFA from the oil occurred via an exothermic process with moderate sorption energy, while the pigment removal via clay occurred via an endothermic process. The result established a Clay-UPPA composite ratio of 70:30 is sufficient to drive the colour removal efficiency towards 80%. The research findings recommend the adoption of UPPA and UPPA-clay composite materials for the refining of vegetable oil and the scale-up of these materials for industrial applications for the improvement of the economy.

## Data Availability

The authors declare that the data used for this research were obtained from laboratory experimentation. The Data stored in a repository will be made available prior to request made to the authors: Mr. Ifeanyi. Ogbu and Prof. Regina Obiageli Ajemba of the Department of Chemical Engineering, Nnamdi Azikiwe University Awka, Nigeria.
